# Platelet count abnormalities and peri-operative outcomes in adults undergoing elective, non-cardiac surgery

**DOI:** 10.1371/journal.pone.0212191

**Published:** 2019-02-11

**Authors:** Isabel A. Weil, Prateek Kumar, Sinziana Seicean, Duncan Neuhauser, Andreea Seicean

**Affiliations:** 1 The Fu Foundation School of Engineering and Applied Science, Columbia University, New York, New York, United States of America; 2 Department of Psychiatry, University of Illinois at Chicago, Chicago, Illinois, United States of America; 3 Department of Medicine, University Hospitals Case Medical Center, Cleveland, Ohio, United States of America; 4 Department of Epidemiology and Biostatistics, Case Western Reserve University, Cleveland, Ohio, United States of America; IRCCS Policlinico S.Donato, ITALY

## Abstract

**Background:**

Anemia and transfusion of blood in the peri-operative period have been shown to be associated with increased morbidity and mortality across a wide variety of non-cardiac surgeries. While tests of coagulation, including the platelet count, have frequently been used to identify patients with an increased risk of peri-operative bleeding, results have been equivocal. The aim of this study was to assess the effect of platelet level on outcomes in patients undergoing elective surgery.

**Materials and methods:**

Retrospective cohort analysis of prospectively-collected clinical data from American College of Surgeons National Surgical Quality Improvement Program (NSQIP) between 2006–2016.

**Results:**

We identified 3,884,400 adult patients who underwent elective, non-cardiac surgery from 2006–2016 at hospitals participating in NSQIP, a prospectively-collected, national clinical database with established reproducibility and validity. After controlling for all peri- and intraoperative factors by matching on propensity scores, patients with all levels of thrombocytopenia or thrombocytosis had higher odds for perioperative transfusion. All levels of thrombocytopenia were associated with higher mortality, but there was no association with complications or other morbidity after matching. On the other hand, thrombocytosis was not associated with mortality; but odds for postoperative complications and 30-day return to the operating room remained slightly increased after matching.

**Conclusions:**

These findings may guide surgeons in the appropriate use and appreciation of the utility of pre-operative screening of the platelet count prior to an elective, non-cardiac surgery.

## Introduction

About 15 million of the 38 million hospitalizations in the United States involve surgery [1–4]. Hospital stays with surgery are roughly 2.5-times costlier than stays without an operation [1–4]. Elective hospital admissions including an operation are 4-times more common than other (non-surgical) elective admissions [1–3]. Given the changing nature of operations that require an admission–in that most patients now have outpatient pre-operative evaluation–more attention is being placed on factors that may predict outcomes.

Identification and amelioration of elements associated with increased risk of peri-operative bleeding is one aim of pre-operative testing. Both anemia and transfusion of blood in the peri-operative period are associated with increased morbidity and mortality across a spectrum of non-cardiac surgeries [5–9]. While tests of coagulation, including the platelet count, have frequently been used to identify patients with an increased risk of peri-operative bleeding, results have been equivocal [5–20].

We examined patients having elective, non-cardiac surgeries–across the spectrum of surgical specialties and levels of operative risk–whose surgery was complex enough to warrant post-operative hospitalization. We used the National Surgical Quality Improvement Project (NSQIP) database [21–24], a large, prospectively-collected, multi-center sample of US adults, over a recent, eleven-year period (2006–2016) to examine the predictive utility of assessing the platelet count in asymptomatic patients undergoing elective surgery for whom remedies for an abnormal platelet count might be available before surgery. First, we wished to quantitate the incidence of the screening platelet count and the frequency of abnormal results immediately prior to surgery. Second, we sought to determine whether patients with platelet count alterations were more likely to receive an erythrocyte transfusion compared to patients with a normal platelet count. Third, we wanted to establish whether a platelet count abnormality, either thrombocytopenia or thrombocytosis, was associated with a higher risk of peri-operative complications or mortality following elective, non-cardiac surgery.

## Materials and methods

### Data source

We used the American College of Surgeons (ACS) National Surgical Quality Improvement Program (NSQIP), a repository of prospectively-collected, clinical data from over 600 community and academic hospitals in the United States [21–24]. Data consists of 273 variables, including demographics, preoperative laboratory values, pre-existing comorbidities, intraoperative variables, and 30-day postoperative morbidity and mortality [21–25]. Hospitals undergo annual inter-rater reliability audits to ensure accurate data collection [21–25]. Data are available for participating institutions from the ACS at https://www.facs.org/quality-programs/acs-nsqip/. All data is fully anonymized by the American College of Surgeons before it is made available for research, including by our team. Because of this prior anonymization, the University of Illinois at Chicago Institutional Review Board waived the requirement for informed consent and approved this study.

### Study population

**Fig 1:**
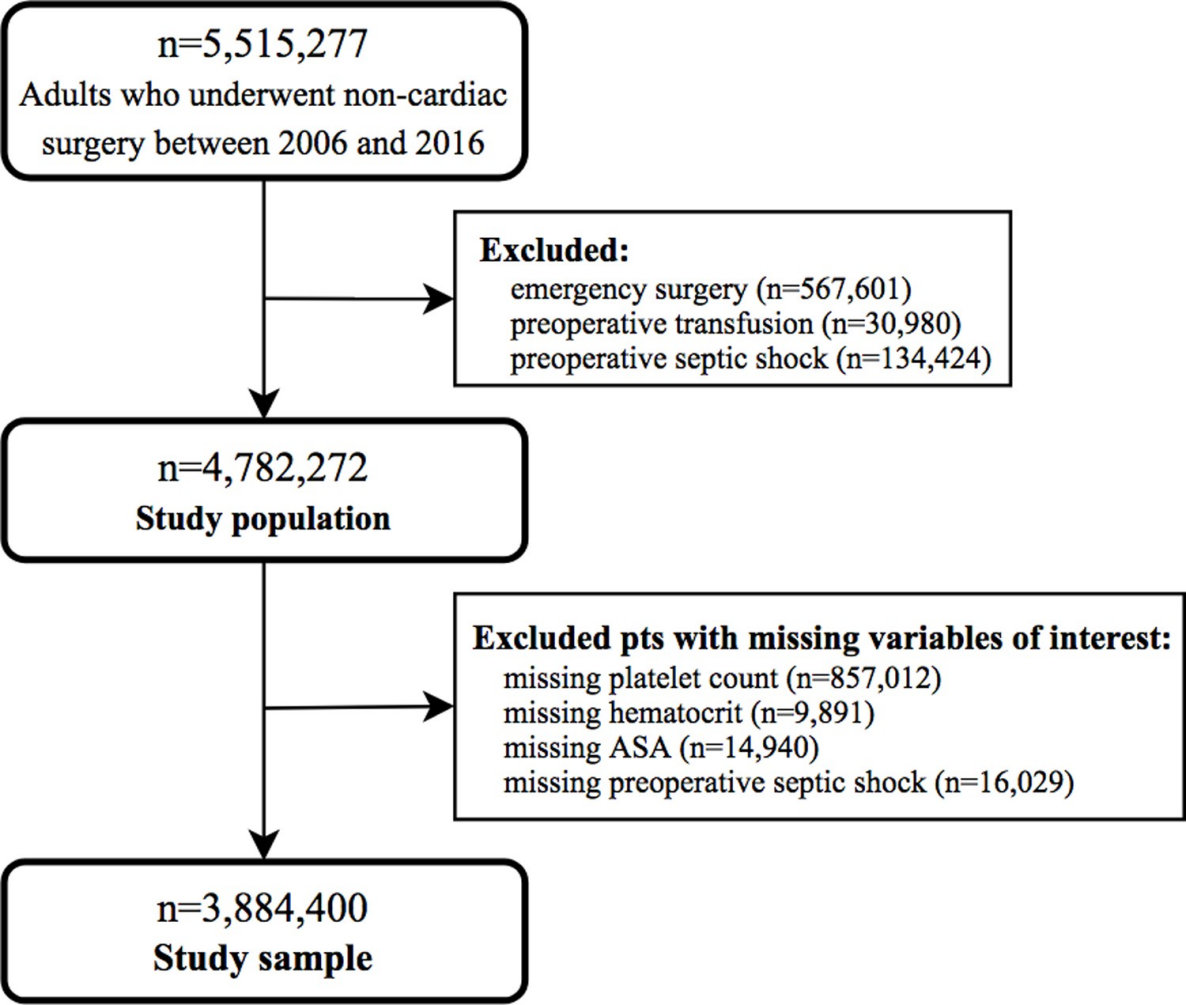
Patient selection criteria. ASA, American Society of Anesthesiologists’ physical status classification.

A total of 5,515,277 patients underwent non-cardiac surgery between 2006 and 2016 (Fig 1). We excluded emergency surgeries (n = 567,601) and patients with preoperative transfusion (n = 30,980) or preoperative septic shock (n = 134,424), features dictating a distinct postoperative course. We excluded patients who had missing values for preoperative septic shock (n = 16,029) or American Society of Anesthesiologists’ (ASA) physical status classification (n = 14,940). We excluded patients who had missing data for our primary variable of interest, platelet count (n = 857,012) and patients with missing data for hematocrit, since hematocrit is importantly related to platelet count (n = 9,891). We did not impute missing baseline values or characteristics. Fig 1 illustrates inclusion and exclusion criteria and determination of the study sample of 3,844,400 patients.

### Platelet categories

A normal platelet count is generally regarded as 150,000 to 450,000 cells per microliter [19]. We segregated the pre-surgical platelet count into these commonly used categories: (1) normal platelet count: 150,000–450,000 platelets/μl; (2) mild thrombocytopenia: 100,000–149,000/μl; (3) moderate thrombocytopenia: 75,000–99,000/μl; (4) severe thrombocytopenia: 50,000–74,000/μl; (5) critical thrombocytopenia: <50,000/μl; and (6) thrombocytosis: greater than 450,000/μl [19,26–28]. NSQIP records the laboratory value(s) most proximate to surgery; if a patient were to receive a transfusion of blood or platelets prior to that blood draw, the effect of that transfusion, if reflected in laboratory testing, would be represented in the laboratory value in NSQIP (21).

### Covariates

We analyzed all available pre- and intra-operative factors previously identified as having an effect on peri-operative outcomes (Table 1) [5–12,14,16–18,20,22,25–40].

**Table 1:** Baseline characteristics stratified by platelet level^a^ (n = 3,884,400).

	Normal	Thrombocytopenia								Thrombocytosis	
	(150-450K), n = 3,569,377	Mild (100-149K) n = 196,339	ASD	Moderate (75-99K) n = 22,013	ASD	Severe (50-74K) n = 9,790	ASD	Critical (<50K) n = 10,305	ASD	(≥450K) n = 76,576	ASD
Age, years, mean ± SD	58 ± 16	65 ± 15	**0.50**	65 ± 14	**0.47**	62 ± 14	**0.31**	58 ± 16	0.05	56 ± 16	0.10
Female	60.1%	34.8%	**0.53**	37.5%	**0.47**	39.3%	**0.43**	52.2%	0.16	66.9%	0.14
Caucasian	75.7%	79.3%	0.08	78.5%	0.06	78.2%	0.04	74.9%	0.00	71.0%	0.09
Admitted from home	97.5%	94.9%	0.13	93.1%	**0.20**	93.5%	0.19	95.3%	0.12	93.3%	0.20
Smoking status											
Never	78.0%	78.3%	0.06	75.2%	0.08	72.5%	0.13	76.0%	0.06	70.5%	0.17
Current	17.9%	16.4%		19.0%		21.8%		18.7%		24.5%	
Previous	4.2%	5.3%		5.8%		5.8%		5.3%		5.0%	
>2 alcoholic drinks per day	2.4%	3.6%	0.07	4.0%	0.09	3.3%	0.06	2.5%	0.01	2.7%	0.02
Partially or fully dependent functional status	2.7%	5.1%	0.13	6.4%	0.18	6.0%	0.16	4.6%	0.10	7.4%	**0.22**
ASA											
1 & 2	53.6%	32.2%	**0.44**	20.7%	**0.73**	17.4%	**0.82**	36.7%	**0.34**	41.9%	**0.24**
3 & 4	46.4%	67.8%		79.3%		82.6%		63.3%		58.1%	
BMI, kg/m^2^, mean ± SD	30.6 ± 8.8	29.1 ± 6.9	0.19	28.7 ± 6.9	**0.23**	29.0 ± 7.2	0.19	30.0 ± 7.9	0.07	28.8 ± 8.5	**0.21**
Diabetes mellitus	15.9%	21.8%	0.15	25.3%	**0.23**	25.5%	**0.24**	18.5%	0.07	19.2%	0.09
Cerebrovascular comorbidities	1.6%	2.5%	0.07	2.5%	0.07	2.1%	0.04	2.0%	0.04	2.0%	0.03
Cardiopulmonary comorbidities	4.5%	6.9%	0.11	8.1%	0.15	7.1%	0.11	5.1%	0.03	6.2%	0.08
Hypertension requiring medication	48.4%	61.3%	**0.26**	61.1%	**0.26**	55.2%	0.14	48.4%	0.00	47.5%	0.02
Renal comorbidities	22.4%	35.4%	**0.29**	38.7%	**0.36**	36.6%	**0.31**	29.0%	0.15	28.5%	0.14
Cancer comorbidities	3.7%	6.4%	0.13	9.8%	**0.25**	10.4%	**0.27**	7.3%	0.16	11.8%	**0.31**
Steroid use for chronic condition	3.3%	5.4%	0.10	8.8%	**0.23**	10.7%	**0.29**	9.5%	**0.25**	7.3%	0.18
Abnormal INR	6.2%	14.5%	**0.28**	25.8%	**0.56**	35.1%	**0.77**	20.2%	**0.42**	14.8%	**0.29**
Anemia	30.2%	40.0%	**0.21**	57.0%	**0.56**	62.9%	**0.70**	49.9%	**0.41**	68.2%	**0.82**
Abnormal LFT	14.7%	22.5%	0.20	35.2%	**0.49**	42.5%	**0.65**	30.6%	**0.39**	39.7%	**0.58**
Abnormal Na	6.3%	8.2%	0.07	11.4%	0.18	13.1%	**0.23**	10.4%	0.15	14.9%	**0.28**
Abnormal WBC count	13.9%	23.2%	**0.25**	39.4%	**0.60**	51.5%	**0.88**	32.1%	**0.44**	31.5%	**0.43**
Multiple CPT codes	37.9%	37.7%	0.00	38.7%	0.02	38.1%	0.00	34.3%	0.07	43.8%	0.12
History indicative of potentially abnormal hemostasis^b^	33.6%	53.1%	**0.40**	69.6%	**0.77**	76.3%	**0.95**	54.1%	**0.42**	55.2%	**0.45**
Prior operation within 30 days	1.8%	2.2%	0.03	2.9%	0.08	3.0%	0.08	2.5%	0.05	6.8%	**0.25**
Resident in OR	58.4%	60.1%	0.03	66.6%	0.17	68.9%	**0.22**	65.4%	0.14	68.0%	**0.21**
General anesthesia	89.5%	88.2%	0.04	90.5%	0.03	92.4%	**0.10**	90.4%	0.03	93.3%	0.14
Surgical specialty											
General surgery	50.6%	48.5%	**0.34**	55.5%	**0.38**	62.7%	**0.39**	59.7%	**0.22**	59.6%	**0.30**
Gynecology	8.1%	2.6%		2.2%		2.2%		4.6%		9.6%	
Neurosurgery	5.1%	4.7%		3.3%		2.6%		4.5%		2.8%	
Orthopedics	18.0%	17.9%		15.7%		13.2%		15.5%		10.4%	
Otolaryngology	2.1%	1.8%		1.5%		1.4%		1.5%		1.3%	
Plastics	2.2%	1.3%		1.0%		1.1%		1.5%		1.8%	
Urology	5.5%	7.7%		5.5%		4.7%		4.1%		3.7%	
Vascular	8.4%	8.7%		12.1%		15.4%		15.5%		10.8%	

ASA, American Association of Anesthesiologists; ASD, Absolute standardized difference; BMI, body mass index; COPD, chronic obstructive pulmonary disease; CPT, Current procedural terminology; DNR, do not resuscitate; LFT, liver function test; ICD, Classification of Diseases; INR, international normalized ratio; Na, sodium; OR, operating room; SD, standard deviation; SIRS, systemic inflammatory response syndrome; WBC, white blood cell. Significant standardized differences (>0.20) are bolded.

^a^Platelet count range is in thousands (K) of cells per microliter.

^b^Patient had one or more of the following risk factors for abnormal hemostasis: history of abnormal bleeding, self-reported family history of bleeding disorders, vitamin K deficiency, currently taking medications that pose a risk for bleeding abnormalities and/or failing to discontinue use of such medications with adequate time for normal hemostasis to be restored, chronic steroid use, chemotherapy and/or radiotherapy for cancer within 90 days prior to surgery, disseminated cancer, renal disease, and/or hepatic disease, abnormal INR.

### Outcomes of interest

The primary outcome of interest was administration of any erythrocyte (RBC) transfusion (either packed RBCs or whole blood), since transfusion of even a single unit of RBCs can impair surgical outcomes [19,20]. NSQIP does not record transfusion of other blood products. Secondary outcomes included: unplanned re-operation or re-admission and death within 30 days of the index surgery; discharge other than to home for patients admitted from home; one or more minor complication; one or more major complication; or a combination of either type of complication (Table 2) [7–9,21,22,24,25,32–40].

**Table 2:** 30-day perioperative complications in the study population stratified by platelet level^a^ (n = 3,884,400).

	Normal	Thrombocytopenia				Thrombocytosis
	(150-450K), n = 3,569,377	Mild (100-149K) n = 196,339	Moderate (75-99K) n = 22,013	Severe (50-74K) n = 9,790	Critical (<50K) n = 10,305	(≥450K) n = 76,576
Perioperative transfusion	4.8%	9.1%	14.6%	16.6%	12.1%	14.4%
Minor postoperative complications	3.6%	4.5%	5.4%	5.4%	4.5%	6.7%
Superficial surgical site infection	1.9%	2.0%	2.5%	2.7%	2.1%	3.6%
Urinary tract infection	1.4%	1.9%	2.2%	2.0%	1.5%	2.3%
DVT or thrombophlebitis	0.5%	0.8%	1.0%	0.9%	1.0%	1.1%
Major postoperative complications	6.3%	9.4%	13.0%	13.1%	10.5%	14.0%
Deep incision surgical site infection	0.6%	0.7%	0.9%	0.9%	0.8%	1.3%
Organ or space surgical site infection	1.1%	1.3%	1.6%	1.7%	1.5%	2.9%
Wound disruption	0.4%	0.5%	0.7%	0.7%	0.5%	1.0%
Pneumonia	0.8%	1.6%	2.4%	2.4%	1.9%	1.9%
Unplanned intubation	0.6%	1.4%	2.3%	2.4%	1.4%	1.3%
>48 hour postoperative ventilator-assisted respiration	0.6%	1.2%	2.2%	2.4%	1.4%	1.2%
Pulmonary embolism	0.3%	0.3%	0.3%	0.3%	0.4%	0.7%
Renal insufficiency or failure	0.4%	0.9%	1.6%	1.8%	1.1%	0.7%
Cerebrovascular accident with neurological deficit	0.2%	0.3%	0.3%	0.2%	0.2%	0.3%
Coma of > 24 hours	0.01%	0.01%	0.03%	0.04%	0.06%	0.03%
Peripheral nerve injury	0.1%	0.1%	0.04%	0.1%	0.1%	0.1%
Cardiac arrest or MI	0.5%	1.1%	1.5%	1.3%	1.0%	0.8%
Graft, prosthesis or flap failure	0.2%	0.2%	0.3%	0.3%	0.3%	0.4%
Sepsis or septic shock	1.4%	2.3%	3.8%	4.3%	2.5%	4.4%
Any infection^b^	4.1%	5.3%	7.5%	8.2%	5.9%	9.9%
Any postoperative complication^c^	8.9%	12.3%	16.3%	16.4%	13.3%	18.0%
Discharged with continued care^d^	8.8%	15.2%	17.7%	16.2%	11.4%	14.5%
30-day readmission^d^	5.6%	8.1%	10.7%	12.0%	8.8%	10.0%
30-day return to the OR	3.0%	4.1%	5.2%	5.1%	4.4%	6.1%
30-day mortality	0.5%	1.5%	3.0%	3.3%	1.9%	1.3%

DVT, deep venous thrombosis; ICD, Classification of Diseases; LOS, Length of Stay; MI, myocardial infarction; OR, operating room; SD, standard deviation.

^a^Platelet count range is in thousands (K) of cells per microliter.

^b^Any ≥1 of: superficial surgical site infection, deep incision surgical site infection, organ or space surgical site infection, and/or sepsis or septic shock.

^c^≥1 minor, major complications.

^d^Data not available prior to 2011.

### Statistical analysis

We compared patients in each abnormal platelet category with patients in the normal platelet count category (Table 1). To control for selection bias, we used propensity matching to appropriately compare patients in each abnormal platelet-level category with patients in the normal platelet-level category; all pre-and intra-operative factors with significant standardized differences were used to generate propensity scores. Propensity score, as applied here, is the probability of a patient being grouped in a specific platelet category given the observed covariates and is determined using multivariate logistic regression, with platelet category as the dependent variable and the significant observed covariates as independent variables [29–31]. Matching by propensity score balances distribution of observed covariates in an observational study similar to randomization in a prospective study [29–31].

To determine propensity scores for each patient based on the variables that were unbalanced, we compared patients with a normal platelet count with those in each abnormal platelet category. To determine covariate balance, we used absolute standardized differences [29–31]. Unlike significance tests, for which statistical differences are reported as p-values, standardized difference does not depend on sample size, which is important in matched analyses because a smaller size of the matched cohort may result in the false notion that matching achieved improved covariate balance [29–31]. Absolute standardized differences of >0.20 were significant [31]. Variables that had absolute standardized differences >0.20 are bolded in Table 1. For example: age, gender, ASA, hypertension requiring medication, renal comorbidities, anemia, abnormal INRs, abnormal WBC count, history indicative of potentially abnormal hemostasis, and surgical specialty were significantly different between patients with mild thrombocytopenia and those with a normal platelet count and therefore were used to generate the propensity score. Using propensity scores to analyze two groups at a time, we matched patients using a 1:1 greedy matching technique [29]. We successfully matched 117,900 of 196,339 mild-platelet-count patients to 117,900 normal-platelet-count patients; 11,894 of 22,013 moderate; 2,156 of 9,790 severe, and 2,584 of 10,305 critical thrombocytopenia patients and 12,555 of 76,576 thrombocytosis patients, including matching exactly by principal Current Procedural Terminology (CPT) (operative) code and primary diagnosis code supporting hospitalization (the International Classification of Disease classification). We confirmed that the baseline characteristics of the patients in the matched sample were similar, and then similar matching was done for each platelet abnormality group.

We describe frequency of 30-day outcomes according to platelet category in the general cohort in Table 2. Logistic regression analysis examined associations between platelet level and adverse outcomes in the general cohort (Table 3). In the matched cohort, conditional logistic regression accounted for the matched nature of data (Table 4). SAS (Version 9.4 SAS Institute) was used for all statistical analyses.

**Table 3:** Association between platelet level^a^ and outcomes of interest in all non-cardiac surgery patients (n = 3,884,400).

	Thrombocytopenia				Thrombocytosis
Outcomes	Mild (100-149K) n = 196,339	Moderate (75-99K) n = 22,013	Severe (50-74K) n = 9,790	Critical (<50K) n = 10,305	(≥450K) n = 76,576
Perioperative transfusion	**1.1 (1.1–1.2)**	**1.3 (1.2–1.3)**	**1.2 (1.2–1.3)**	**1.2 (1.1–1.2)**	**1.1 (1.1–1.2)**
Minor complications	**1.2 (1.2–1.3)**	**1.5 (1.4–1.6)**	**1.5 (1.4–1.7)**	**1.3 (1.1–1.4)**	**1.9 (1.8–2.0)**
Major complications	**1.5 (1.5–1.6)**	**2.2 (2.1–2.3)**	**2.2 (2.1–2.4)**	**1.7 (1.6–1.9)**	**2.4 (2.4–2.5)**
Any complications	**1.4 (1.4–1.5)**	**2.0 (1.9–2.1)**	**2.0 (1.9–2.1)**	**1.6 (1.5–1.7)**	**2.3 (2.2–2.3)**
Discharged with continued care^b^	**1.9 (1.8–1.9)**	**2.2 (2.1–2.3)**	**2.0 (1.9–2.1)**	**1.3 (1.2–1.4)**	**1.7 (1.7–1.8)**
30-day readmission^b^	**1.5 (1.5–1.5)**	**2.0 (1.9–2.1)**	**2.3 (2.2–2.5)**	**1.6 (1.5–1.8)**	**1.9 (1.8–1.9)**
30-day return to OR	**1.4 (1.3–1.4)**	**1.8 (1.7–1.9)**	**1.7 (1.6–1.9)**	**1.5 (1.3–1.6)**	**2.1 (2.0–2.1)**
30-day mortality	**2.9 (2.8–3.0)**	**6.1 (5.7–6.6)**	**6.8 (6.0–7.5)**	**3.9 (3.4–4.5)**	**2.5 (2.4–2.7)**

CPT, current procedural terminology; LOS, length of hospital stay; OR, operating room.

^a^Platelet count range is in thousands (K) of cells per microliter.

^b^Data not available prior to 2011.

**Table 4:** Association between platelet level^a^ and outcomes of interest in all non-cardiac surgery patients after matching (n = 294,178).

	Thrombocytopenia				Thrombocytosis
Outcomes	Mild (100-149K) n = 117,900	Moderate (75-99K) n = 11,894	Severe (50-74K) n = 2,156	Critical (<50K) n = 2,584	(≥ 450 K) n = 12,555
Perioperative transfusion	**1.2 (1.1–1.2)**	**1.4 (1.3–1.5)**	**1.5 (1.2–1.7)**	**1.6 (1.4–1.9)**	**1.4 (1.3–1.5)**
Minor complications	1.0 (0.9–1.0)	0.9 (0.8–1.0)	0.8 (0.7–1.0)	1.0 (0.8–1.2)	**1.2 (1.1–1.3)**
Major complications	1.0 (1.0–1.0)	1.0 (1.0–1.1)	0.9 (0.8–1.0)	1.1 (0.9–1.2)	**1.2 (1.2–1.3)**
Any complications	1.0 (1.0–1.0)	1.0 (0.9–1.1)	0.9 (0.8–1.0)	1.0 (0.9–1.2)	**1.2 (1.2–1.3)**
Discharged with continued care^b^	1.0 (1.0–1.1)	1.0 (0.9–1.0)	0.7 (0.5–1.0)	0.9 (0.6–1.2)	1.2 (1.0–1.4)
30-day readmission^b^	1.0 (1.0–1.1)	1.1 (1.0–1.2)	1.2 (0.8–1.6)	1.2 (0.8–1.6)	1.1 (0.9–1.3)
30-day return to OR	1.0 (1.0–1.0)	0.9 (0.8–1.0)	0.8 (0.6–1.0)	0.9 (0.7–1.2)	**1.3 (1.2–1.4)**
30-day mortality	**1.2 (1.1–1.3)**	**1.6 (1.3–1.8)**	**1.6 (1.2–2.1)**	**1.9 (1.4–2.5)**	0.8 (0.7–1.0)

CPT, current procedural terminology; LOS, length of hospital stay; OR, operating room. All odd ratios are compared to normal platelet levels. Odd ratios that are significant are bolded.

^a^Platelet count range is in thousands (K) of cells per microliter.

^b^Data not available prior to 2011.

## Results

Among the 5,515,277 patients in the ACS-NSQIP database comprising our global population of patients who underwent non-cardiac surgery from 2006–2016 (Fig 1), 733,005 were excluded because they had emergency surgery, a pre-operative transfusion, or septic shock. This left 4,782,272 patients undergoing elective surgery; 82.1% of this group had at least one platelet count performed prior to the index surgery. Of the 4,782,272 patients, 897,892 were excluded because of missing clinical variables of interest, leaving our study sample of 3,884,400 patients who underwent an elective, non-cardiac surgery in one of eight specialties (Fig 1 and Table 1). Approximately 51% were general surgery patients and nearly 18% were orthopedic surgery patients; remaining groups were: vascular surgery (8.4%), gynecology (8.1%), urology (5.5%), neurosurgery (5.1%), plastic surgery (2.2%), and otolaryngology (2.1%) (Table 1). Approximately 92% of the study population had a normal platelet count; mild, moderate, severe, and critical thrombocytopenia were seen in 5%, 0.6%, 0.3%, and 0.3% of patients, respectively; 2% had thrombocytosis (Table 1). We compared baseline pre-operative characteristics and two intraoperative variables (resident in the OR as a surrogate marker of a teaching hospital; and multiple Current Procedural Terminology (CPT) codes at index surgery) between patients with abnormal (thrombocytopenia or thrombocytosis) and normal platelet counts; we identified several standardized differences between groups (significant values, >0.20, bolded in Table 1).

We considered intra- and post-operative outcomes (under the rubric of 30-day peri-operative complication rates) in the four thrombocytopenia groups and the thrombocytosis group. Rates of nearly all minor and major complications, any complications in aggregate, discharge with continued care, unscheduled readmission or unplanned return to the operating room and mortality within 30 days were higher in every subgroup, when compared, as a uniform group, against the normal platelet count population (Table 2). In the mild thrombocytopenia group, complications were more frequent in 23 of 26 peri-operative outcome groupings. Similarly, in the moderate thrombocytopenia group, all 26 outcome groups showed complication rates greater than the normal population; in the severe thrombocytopenia group, 24 of 26 were greater; in the critical thrombocytopenia groups, 25 of 26 categories were higher; and in the thrombocytosis population, 25 of 26 outcome categories had greater complication rates compared to that of the normal platelet population (Table 2).

We assessed potential associations between pre-operative platelet count and outcomes of interest for patients with a platelet count abnormality, as compared to counts and outcomes for patients with a normal platelet count (Table 3; 8 outcomes of interest are examined). For all categories of thrombocytopenia as well as the thrombocytosis group, complication rates were significantly higher than in the normal-platelet-count group.

To align patients in a manner as closely as possible and minimize selection bias, we used propensity matching to assign patients from each abnormal platelet count category with patients from the normal platelet count category, also matching exactly by surgery type using the principal diagnosis and primary operative procedure. All pre-and intra-operative factors with significant standardized differences (Table 1) were used to generate propensity scores. We matched patients using a 1:1 greedy matching technique (Table 4); outcomes of interest remained the same as in the general cohort. Across all categories of thrombocytopenia, only rates of perioperative transfusion and mortality remained significant after matching. Odds ratios and 95% confidence intervals for receiving perioperative transfusion were: 1.2, 1.1–1.2 (mild thrombocytopenia), 1.4, 1.3–1.5 (moderate thrombocytopenia), 1.5, 1.2–1.7 (severe thrombocytopenia), and 1.6, 1.4–1.9 (critical thrombocytopenia). Odds ratios and ranges for mortality were: 1.2, 1.1–1.3 (mild thrombocytopenia), 1.6, 1.3–1.8 (moderate thrombocytopenia), 1.6, 1.2–2.1 (severe thrombocytopenia), and 1.9, 1.4–2.5 (critical thrombocytopenia). Finally, in the thrombocytosis group, the risks of transfusions, 30-day return to OR, and major, minor, or any complications were all slightly increased compared to the normal platelet count patients (Table 4). Unlike with thrombocytopenic patients, the risk for 30-day mortality for patients with thrombocytosis was not significantly different than that in patients with normal platelet counts (OR 0.8, CI 0.7–1.0).

## Discussion

### Overview

We studied a large sample of five and a half million patients who underwent elective, non-cardiac surgery to examine the predictive value of assessing the platelet count prior to surgery. Of the 4,782,272 patients eligible for analysis over an eleven-year period from 2006 through 2016, 82.1% had a least one platelet count performed prior to the index surgery. After excluding patients with missing variables of interest (see Fig 1), the study sample comprised 3,844,400 patients, of whom 91.9% had a normal platelet count (Table 1). Of those with abnormal platelet counts, 5.1%, 0.6%, 0.3%, and 0.3% had mild, moderate, severe, and critically low platelet counts prior to elective surgery; 2.0% had thrombocytosis (Table 1). Compared to patients with a normal platelet count, those with thrombocytopenia and thrombocytosis were more commonly general surgery patients rather than one of the other seven specialties, a feature particularly evident with increasing thrombocytopenia. In addition, patients with both types of platelet count abnormalities (Table 1) more often presented with one or more characteristics for an increased risk for peri-operative morbidity than patients with a normal platelet count. Their increased risk was further substantiated in the next analysis, where, in the first 30-days following the index surgery, nearly all minor, major, and any complications as an aggregate, discharge with continued care, unplanned hospital re-admission or unplanned return to the operating room, as well as mortality rates, were higher in every group (Table 2). Using our eight outcomes of interest to compare patients with thrombocytopenia or thrombocytosis to patients with a normal platelet count, odds ratios for complications are higher in every group of thrombocytopenia or thrombocytosis (Table 3). To ensure that these differences were not explained by differences in composition of the groups (Tables 1 and 2) and to minimize selection bias (Table 3), we used propensity matching to pair patients from each platelet group with a patient from the normal platelet group, including matching by specialty type (e.g., gynecology patient with gynecology patient), principal diagnosis (ICD-9 code), and primary procedure performed (CPT code), using a 1:1 greedy matching technique. The odds for each of two critical outcome features, peri-operative transfusion and 30-day mortality, remained increased in all thrombocytopenia groups; odds for all other outcomes were not affected (Table 4). In the thrombocytosis group, the odds of perioperative transfusion, complications (major/minor/any), and 30-day return to OR remained slightly increased. Thrombocytosis did not affect odds for 30-day mortality (Table 4).

### Clinical implications

While traditionally, preoperative laboratory evaluation was nearly universal, recent work suggests that laboratory screening is rarely useful in the absence of explicit historical or examination risk factors [10–20,39]. The American College of Physicians and the American Society of Anesthesiologists recommend against routine laboratory testing [13,14]. Several groups have demonstrated that not only do coagulation alterations affect only a small fraction of the surgical population, but the history and physical exam are equally capable of predicting an abnormal laboratory test and neither testing nor the history and physical examination optimally predict surgical outcomes [13–18,39].

Nonetheless, pre-operative testing remains common. In this study of patients undergoing elective, non-cardiac surgery, for whom history and physical exam preceded surgeries–and for whom one might expect medical condition(s) could be optimized prior to surgery in nearly all cases–we found that most subjects (82.1%) had a platelet count obtained. In nearly all patients (91.9%), the platelet count was normal; and, while patients with a platelet count alteration appear to have more peri-operative complications than those with a normal count, when one matches patients carefully, differences in outcomes are more limited. Besides rates of transfusion, only rates of mortality remain affected by thrombocytopenia while six other complications of interest do not; rates of major/minor complications and return to OR remain affected by thrombocytosis, but rates of other morbidity and mortality do not.

Single center studies of discrete surgical populations have shown that thrombocytopenia and thrombocytosis tend to be variably associated with increased morbidity [40–43]. Furthermore, transfusion of platelets may be useful in specific patients undergoing an invasive procedure; the American Association of Blood Banks (AABB) has made four active or positive recommendations in patients with platelet counts from 10-50K/μl, and two negative recommendations, although they recognize that in some situations, such as cranial surgery, a higher platelet count may be needed [19].

Glance and colleagues examined a subset of 316,644 patients from NSQIP, with no identified indications for laboratory testing, undergoing non-cardiac surgery in 2008–2009 [20]. Using univariate logistic regression, they showed that patients with mild thrombocytopenia (platelet count of 101-150K/μl), moderate and severe thrombocytopenia (all patients with counts <101K/μl grouped), or thrombocytopenia (>450K/μl) had increased rates of post-operative transfusion (7.3%, 11.8%, and 8.9%, respectively, compared with 3.1% in normal) and 30-day mortality (1.5%, 2.6%, 0.9%, respectively, compared with 0.5% in normal count patients) [20]. In multivariate analysis, both groups of thrombocytopenia patients and those with thrombocytosis had elevated odds of a blood transfusion compared to the normal cohort; mortality was higher in both thrombocytopenia groups, but the odds ratio crossed 1.0 in thrombocytosis patients [20]. In our study, which includes more than ten times as many patients and more recent data, we examined multiple subtypes of patient with thrombocytopenia along the lines of the AABB clinical practice guidelines [19–20]. We did not impute values for missing preoperative variables of interest; we controlled for the presence of history or examination features that suggest a potential platelet disorder; and we included only elective cases, so as to include patients in whom a medical condition, including platelet abnormalities, could be rectified pre-operatively. Furthermore, Glance et al categorized by broad CPT category [19], which has limitations: for example, CPT range of 60000–60999 (“endocrine system”) includes thyroidectomy with a transthoracic approach (60270), non-laparoscopic adrenalectomy (60540), and resection of a carotid body tumor (60600), each of which is quite distinct. In contrast, we matched patients from each study group with a control patient by principal CPT code and primary diagnosis code. In Glance et al, only peri-operative transfusion and 30-day mortality were outcomes of interest, compared to our broader range of outcomes [20]. Finally, Glance and colleagues did not use propensity matching, which minimizes selection bias by randomly assigning a study patient with a normal cohort patient [20,29–38]. The present analysis represents the largest and most comprehensive analysis of platelet count abnormalities and peri-operative outcomes performed, with implications for everyday care of the surgical patient.

### Limitations

This study has limitations. Although NSQIP data collects patient-level clinical data prospectively, the present report is retrospective in nature; one cannot unequivocally establish cause-and-effect relationships. NSQIP is a surgical database; patients who were not offered surgery or declined surgery are not included, which may introduce selection bias. In spite of matching on propensity scores, we cannot exclude the possibility that there are differences between patients in each platelet category with preoperative factors for which we lack data; we included all variables in NSQIP that may have an influence on outcome, a broad collection of pre- and intra-operative variables [5–12,14,16–18,20,22,25,32–40]. Our study focuses on the 30-day perioperative period; NSQIP does not contain longer follow-up. Institution-specific data, including data on clustering to show patients operated on by the same surgeon or at the same institution, are not included in NSQIP. We cannot discern reasons for readmission because NSQIP started to collect this data only in 2011. As participation in NSQIP is voluntary, NSQIP may not recapitulate the US population of patients who undergo elective, non-cardiac surgery requiring hospitalization. However, the gender and race distributions in the NSQIP database are consistent with the overall US population; and, all of the data is collected prospectively from a number and variety of institutions, which provides a large and diverse sample size [22]. NSQIP data are collected in a standardized manner, with annual quality checks, and data reporting achieves >95% 30-day outcome follow-up rates across consecutive cycles, accurately and reproducibly [20,22,24,25]. NSQIP is an effective database to analyze surgical quality and outcomes; it becomes more reliable as the sample sizes increase and becomes more capable of predicting major events such as perioperative transfusion, death, or unplanned return to the OR, outcomes which were at the center of this investigation [20,22,24,25].

## Conclusions

While pre-operative laboratory evaluation of the platelet count in patients who undergo elective, non-cardiac surgery is common, abnormalities in platelet counts are not. When patients with apparent thrombocytopenia are matched carefully with normal-platelet-count patients based on a variety of peri-operative factors and are matched exactly by principal procedure and primary diagnosis underlying their surgeries, most of the differences in terms of complications–including minor or major complications, discharge to any place other than one’s home, unplanned or unscheduled operation or re-admission within 30 days–are no longer significant. However, the odds of perioperative transfusion and 30-day mortality remain increased, particularly for patients with moderate, severe, and critical thrombocytopenia. On the other hand, for patients with apparent thrombocytosis upon pre-operative evaluation, once matched appropriately as above, there is no change in risk of 30-day mortality, but risk of transfusion remains increased and risk for major or minor complications also remains slightly increased. These findings may guide surgeons and others to better appreciate the utility of pre-operative screening of the platelet count prior to an elective, non-cardiac surgery.
